# Abnormal Secretion

**Published:** 1873-01

**Authors:** O. Willson


### ARTICLE II.

### *Abnormal Secretion.*

BY DR. O. WILLSON.

Read before the Illinois State Dental Society.

With that depression of spirits which occasionally creeps over the dental operator upon a review of his work, and with that feeling of tremendous necessity crowding upon us to know more of the causes destructive of the dental apparatus, we commenced, last fall, a record of observations, keeping it in tabular form, thus:

Age of patient, sex, condition of body, color of decay, time after eating, and general character of the saliva.

That record is growing—has been of some interest to us—but we regret, gentlemen, our inability, at this time, to draw from it conclusions exact enough to be much edifying to this honorable body. Yet since the design of this paper is not so much to teach as to solicit others to push the investigation; not so much to tell what we do know as what we don't; it may not be amiss to say that secretion is the separation of the materials of the blood by the animal economy, and upon the natural performance of this, together with the excretory functions, depends the health of the body; provided the proper supply of material from without be maintained.

It is either glandular or exhalant. If the gland be simple it is termed a follicle. Follicular is the general character of mucous secretions throughout the body.

Occasionally it is a function of the excretory system to protect the body; so also with the secretory to nourish; though at present it is difficult to tell when the office of one ends and the other commeaces, such is the wonderful harmony of the parts.

If the excretory functions become abnormal, warning is not tardy, the cause not often obscure, and the remedy is patent. Not so with the secretory. Subtle, obscure, and sublime in its design, it continues its silent work, almost defying, like the nervous system, investigation complete. If normal, it itself is almost a synonym for happiness. If abnormal, there are but two classes of persons—patients and medical gentlemen—to whom this investigation is as important as to us, who are daily battling with agents destructive to the dental apparatus, for the two-fold purpose of honorable acquisition, and to a gratifying extent, alleviating the ills of our kind.

It is of paramount importance, that we, as dentists, ascertain the source and character of these agents, that remedies may be multiplied and placed within the easy reach of all, for vigorous exercise.

It avails little that we warrant our work—at best a low type of professional standing—for the question immediately occurs, what is its commonly accepted interpretation. Whatever construction we ourselves put upon it, our patients expect the arrest of decay and immunity from pain. The guarantee will not add to the conscientious dentist's present stock of skill, or to any one's honesty, and if the latter be absent, better would it be for all concerned that the relation of patient and dentist cease at once; and in the exercise of the former, it ought not to be expected that filling a tooth will make it better than other teeth in the same mouth, and if it endure as long without decay as they do, this to our mind is the limit of requirement.

And yet it is mortifying in the extreme, after we have done our best to reclaim the health of the dental organs, to see here and there the gradual but sure encroachment of the enemy, after having as we believe cleaned thoroughly, impacted perfectly, and finished beautifully, to notice the approach of decay, or to see the supporting tissues lose vitality and recede; and unless there are some present like the dentist just around the corner, who fills teeth so "they last forever," we think there may be dentists here who sympathise with this the writer's confession.

Dental caries we believe to be on the *increase*—is now well nigh universal—while recession of supporting tissue by mechanical irritation and other causes is fearfully prevalent, even with the young.

What is the cause of this destruction, and the remedy what, are questions as pertinent now, perhaps, as at any time previous.

"Sins do not spring out of the ground," neither is caries from within, else the more we excavate the worse would we make it. The recuperative power is so low that the teeth *submit* to the process of filling; hence also the unheard of phenomenon of a carious tooth restoring itself.

But we *submit* that our present treatment is too topical.

In health of the moisture of the mouth, that secreted by the parotid glands—conglomerate—is a clear, limpid fluid, slightly alkaline. That from the submaxillary and sublingual—also conglomerate—is slightly alkaline, but is viscid or ropy. The follicular secretion, or the moisture of the mucous membrane at or near the fauces abounds in this viscous albuminous material, and at the margins of the gums is, we believe, a little acidulate, since litmus is reddened at these points more commonly than we find it elsewhere, after making due allowance for any foreign deposit.

If this be correct, the equipollence of these two directly opposite substances, in health of the oral cavity, is beautiful, each in turn rushing to the rescue of the teeth as the enemy of the one or the other enters the chemical laboratory.

In health of these well-springs, and when no suggestive touch, odor or mental impression is made, the whole seems neutral, and not very destructive to food; and unless there be some defect in the formation of enamel at depressions, teeth, even those wanting in the best proportion of phosphates and animal material, are not liable to extensive decay in this condition.

But extensive destruction is the *rule*—the reverse is the exception, and the destroyer of teeth is from without.

A diseased body it is natural to suppose would involve an abnormal condition of the mouth; and an unhealthy mouth it would seem pre-supposes a general derangement of the body. But though the condition of the one does effect the other in many instances, yet the former conclusions are not fully warranted by our own observations. One reason for this may be that secretion is nearly as variable in character as in locality. Even follicular secretion from so similar organs is very variable in ear, nose, fauces, stomach and intestines, and whether the gland be follicular, conglobate or conglomerate, one may be diseased for life even, and others remain normal in function.

Close observers tell us that 100 lbs. of phosphates, carbonates and other bone material on the average are by abnormal secretion and excretion thrown away from the body annually. And yet with this enormous loss and tax on some of the organs, others for years remain *intact.* Such are the wonderful resources of the body.

Notwithstanding these glaring possibilities, the fact must still remain that abnormal secretion is constitutional as a general proposition, and must ultimately be treated as such.

That the secretions of the mouth are generally abnormal is we think the conviction of most persons called to dispute the progress of dental caries. Ostensibly these destructive agents are either an excess of acid—the commonly accepted opinion—or of alkali.

At first it is odd to suppose that alkali, an object so near akin in substance to dentine, two-thirds of which is phosphate and carbonate, but remembering that its animal portion is protein, (mostly albumen fibrin and casein,) and that alkali forms with it soluble compounds, it seems to us that the conclusion, that under such condition the dissolution of dentine is inevitable, is just as logical as that acid neutralizes alkali, which itself, by the way, must be taken with some degree of allowance.

The prevalence of large deposits of salivary calculus, and the fact that once deposited in a tooth it effectually arrests decay, while the inference is strong that alkali in the secretion is largely in excess, might lead to the erroneous conclusion that if alkali would destroy teeth at all it would be in such instances.

But tartar is not exclusively calcareous; it is a concretion of broken down epithelium, fibrin and quite a variety of precipitates, including phosphates. Acids will not always dissolve it, though sometimes this is due to the fat it contains.

Tartar is more a residuum of some chemical union rather than a cause of itself. Let us not deceive ourselves by giving alkali a less prominent place in this destructive process than it deserves. Its action is more remote in inception, but not consequently less effective.

We commenced observations in the ordinary way; by using the litmus paper, an article that should be much used by every dentist, and since a good article is not always easily obtained, allow us to give Caldwell's method of making it.

Digest litmus—obtainable at drug stores—with six parts of water; filter, divide half of the filtrate into two equal parts, and carefully saturate the free alkali in one of these parts with sulphuric acid until the liquid has taken a red color that does not disappear after standing a few minutes; add the other part to this, color strips of unsized paper in the blue liquid and dry them. Add sulphuric acid to the other half of the litmus until a permanent red color is just obtained; color strips of unsized paper in this solution, dry and keep both in a dark place. Acid turns the blue paper red, and alkali turns the red paper blue.

At first we were much elated with the information this test gave; acid reaction in nearly every instance for a number of days; soon there was none. Concluding that if there was no acid reaction there must be an alkaline, for caries was not less abundant, what was our surprise, often to find that there was reaction by neither test, and the decay seemingly as rapacious as when an acid or alkaline reaction was plainly observable. Then came to us the knowledge—common it may be to you—that litmus is a test for free acids or alkali only, while either may be secreted in abnormal quantities, driving on their silent work of destruction, just as soon as an affinity comes along to cause a divorce of the neutral compound. This leads to closer analysis; for while it is highly important that we know if a free acid or alkali be present, it is important to know the general character of the acid, and if it be an alkali, whether it be carbonaceous, for acid acts readily upon alkalies containing carbon, but will not, for instance, on sal ammonia—an alkali; it will, however, if the ammonia be made a carbonate.

If these two conditions be necessary knowledge, it is still more so, to find out the chemical cause, behind the curtain of a neutral sulphate, nitrate or carbonate, &c., and towering above all is the important question of whence and from what cause this abnormal condition. Would it not be wise to imitate our medical brethren, and go to the urine, perspiration, &c., for analysis, detection and suggestion.

While testing with the litmus it is indispensable that the fingers should not touch it, and that it be not laid upon wood while moist, or anything that will color it as these will.

It is possible, we believe, to carry an analysis of this kind too far; beyond the organic vitalized condition into original elements, which themselves are part and parcel of necessary bodily unity or health.

Water, ammonia and carbonic acid, says Liebig, are the ultimate products of decay and fermentation, yet these same substances contain the elements necessary for the support of both animal and vegetable life.

The analysis should isolate the organic substances without decomposing them.

All substances that irritate the stomach, produce acid in the mouth.

Iodine, bromine or mercury, secreted by the saliva, occasioned by medicines, can usually be detected by tests, applied directly. Not so with organic material. The difficulty lies in the evaporation of salival or alcoholic solution, of obtaining a saliva residuum without decomposition. Saliva, or solutions of it, will soon change in the atmosphere by putrefaction.

One of the most desirable tests, doubtless, especially for alkali, is the observations of the crystals or lime salt after evaporating in alcoholic extract of saliva. Lactic acid can thus also be detected in pathological saliva. This requires, of course, a knowledge of crystalology and microscopy, of which the writer is comparatively ignorant at present.

We incline to the opinion that the occurrence of free acid in the mouth, like excessive acidity of the stomach or intestines, the uric acid of gout, the lactic acid of rheumatism, the oxalic acid of oxalurea, and the deposition of urates in the urine, is caused by defective pulmonary oxydation in a large number of cases.

These acids are intermediate compounds, to some extent normal in the system, but ought never to be secreted as such; should be oxydized for excretion, as carbonic acid and water. Their secretion occurs to those who eat freely and breathe lightly. Sometimes debility prevents pulmonary oxydation, even with out-door exercise.

Prof. Prescott thinks that a good substitute for air by the lungs is nitro-muriatic acid, and the chlorate or permanganate of potassa. Free water drinking also favors oxydation.

We are conscious, gentlemen, that this is more discussion and inquiry than information; but such is science.

